# Impact of Polypharmacy on Candidate Biomarker miRNomes for the Diagnosis of Fibromyalgia and Myalgic Encephalomyelitis/Chronic Fatigue Syndrome: Striking Back on Treatments

**DOI:** 10.3390/pharmaceutics11030126

**Published:** 2019-03-18

**Authors:** Eloy Almenar-Pérez, Teresa Sánchez-Fito, Tamara Ovejero, Lubov Nathanson, Elisa Oltra

**Affiliations:** 1 Escuela de Doctorado, Universidad Católica de Valencia San Vicente Mártir, 46001 Valencia, Spain; eloy.almenar@ucv.es (E.A.-P.); mt.sanchez@ucv.es (T.S.-F.); 2 School of Medicine, Universidad Católica de Valencia San Vicente Mártir, 46001 Valencia, Spain; tamara.ovejero@ucv.es; 3 Kiran C Patel College of Osteopathic Medicine, Nova Southeastern University, Ft Lauderdale, FL 33314, USA; lnathanson@nova.edu; 4 Institute for Neuro Immune Medicine, Nova Southeastern University, Ft Lauderdale, FL 33314, USA; 5 Unidad Mixta CIPF-UCV, Centro de Investigación Príncipe Felipe, 46012 Valencia, Spain

**Keywords:** fibromyalgia (FM), myalgic encephalomyelitis/chronic fatigue syndrome (ME/CFS), microRNA, miRNome, pharmacogenomics, pharmacoepigenomics, SM2miR, Pharmaco-miR, repoDB, ME/CFS Common Data Elements (CDEs)

## Abstract

Fibromyalgia (FM) and myalgic encephalomyelitis/chronic fatigue syndrome (ME/CFS) are diseases of unknown etiology presenting complex and often overlapping symptomatology. Despite promising advances on the study of miRNomes of these diseases, no validated molecular diagnostic biomarker yet exists. Since FM and ME/CFS patient treatments commonly include polypharmacy, it is of concern that biomarker miRNAs are masked by drug interactions. Aiming at discriminating between drug-effects and true disease-associated differential miRNA expression, we evaluated the potential impact of commonly prescribed drugs on disease miRNomes, as reported by the literature. By using the web search tools SM2miR, Pharmaco-miR, and repoDB, we found a list of commonly prescribed drugs that impact FM and ME/CFS miRNomes and therefore could be interfering in the process of biomarker discovery. On another end, disease-associated miRNomes may incline a patient’s response to treatment and toxicity. Here, we explored treatments for diseases in general that could be affected by FM and ME/CFS miRNomes, finding a long list of them, including treatments for lymphoma, a type of cancer affecting ME/CFS patients at a higher rate than healthy population. We conclude that FM and ME/CFS miRNomes could help refine pharmacogenomic/pharmacoepigenomic analysis to elevate future personalized medicine and precision medicine programs in the clinic.

## 1. Introduction

Fibromyalgia (FM) is a debilitating disorder characterized by a low pain threshold and muscle tenderness accompanied by bowel abnormalities, sleep disturbances, depressive episodes, cognitive problems, and chronic pain [1,2,3,4]. Though commonly comorbid with myalgic encephalomyelitis/chronic fatigue syndrome (ME/CFS), a disease also showing a complex clinical pathophysiology [5,6,7,8,9,10,11], these syndromes have been classified by the International Classification of Diseases, Tenth Revision, Clinical Modification (ICD-10-CM), with separate codes (M79.7 and R53.82 or G93.3 if post-viral, for FM and ME/CFS, respectively) [12]. However, disease distinctions remain under debate [5]. 

Although possibly underestimated, the global prevalence for FM has been set at 2–8% and at 0.23–0.41 for ME/CFS with predominant ratios of females over males [13,14,15,16,17]. In addition, increasing numbers of patients being affected at early ages [18] highlights the considerable and raising needs for appropriate healthcare programs and the stepping demands for the alleviation of associated economic/social burdens. 

Post-exertional malaise (PEM), a clinical hallmark of ME/CFS, together with additional clinical and biological parameters differing between these two diseases [19,20,21,22,23,24] seem to support a distinct underlying pathophysiology and possibly etiology for FM and ME/CFS. Aimed at clarifying this diagnostic conflict through an improved understanding of the biology of disease onset and evolution, some research groups, ours included, have set out to identify molecular biomarkers of these illnesses [25].

MicroRNAs or miRs constitute attractive candidates for the diagnosis of FM and ME/CFS, as they have been found to associate with the disease state of other complex chronic diseases [26,27] and may even be used to measure disease stage and response to treatments [28]. In their mature form (20–22 nts), they epigenetically control gene expression by directing particular sets of mRNAs, presenting partial complementation in their 3’UTRs, to degradation [29]. Other regulatory mechanisms have also been linked to the activity of these small molecules [30]. 

In addition to their biomarker value, miRNAs could potentially be targeted by small drugs, either directly through the binding of chemical compounds to particular grooves or pockets of their secondary structures, in their mature or precursor forms, as isolated or complexed molecules, or indirectly by interfering with proteins involved in their biogenesis or recycling, including regulation of transcription factors driving miRNA synthesis [31,32,33,34]. Therefore, directional FM and ME/CFS treatments based on miRNA targeting strategies are envisioned as potential curative therapies by themselves or as co-adjuvants in the near future.

MiRNA capacity to sense and respond to environmental cues [35,36,37], however, makes the establishment of correlations between particular disease states and miRNA profile changes challenging. To minimize potential environmental confounding factors, healthy participants are often population-matched by sex, age, and quite frequently BMI (body mass index) with the participating patient group. Careful selection of participants and proper study design are key factors in identifying miRNA disease-associated profiles (disease miRNomes), as miRNA levels also change in response to hormone challenges, during aging and metabolic states [38,39,40,41], including the post-prandial estate [42]. In the context of FM and ME/CFS, since miRNomes change with exercise [43], inclusion of sedentary control groups would be desirable.

Current treatments of both FM and ME/CFS diseases are symptom-palliative only [44,45,46,47,48]. Due to multi-symptomatology, patient prescriptions frequently involve polypharmacy, which may significantly impact downstream molecular analysis of the disease. With this perspective, a recent joint initiative worked out by the NINDS (National Institute of Neurological Disorders and Stroke) at the NIH (National Institute of Health) in Bethesda, MD (USA) and other federal agencies has made available case report forms (CRFs) and guidelines to register drug use in ME/CFS studies [49]. The ME/CFS Common Data Elements initiative or CDE Project aimed at standardizing clinical relevant variables for the study of ME/CFS covers various areas organized by domains and sub-domains. Information is publicly available at the NINDS Common Data Elements web page [49]. 

A recently observed feature of miRNAs is their role in determining drug efficacy [50,51]. The traditional field of pharmacogenomics dealing with how individual genomic features, including SNPs (single nucleotide polymorphisms) and CNVs (copy number variants), influence a patient’s response to drug-based treatments and sensitivity to toxic effects is becoming complemented by individual epigenetic profiles including alternative splicing events and miRNomes (pharmacoepigenomics), with the aim of elevating predictions of the most effective and safest options towards improved personalized treatments in the clinic [52,53,54,55]. In addition to epigenetic regulation of drug targets, regulation of genes related to drug absorption, distribution, metabolism, and excretion (ADME) may translate into significant inter-individual differences to drug response [56]. In this context, it should be of relevance to take into account a patient’s FM or/and ME/CFS condition when standardized treatments for diseases other than FM and ME/CFS are in need. In particular, FM and ME/CFS associated miRNA profiles might promote drug efficacy or inhibit drug function when compared to non-FM and non-ME/CFS patients and consequently impact or influence an FM and ME/CFS patient’s response to pharmacological treatments or sensitivity to adverse reactions. Interestingly, and in line with this, FM and ME/CFS patients report suffering from multiple chemical sensitivity [57].

In this paper, we have interrogated the potential impact of commonly prescribed drugs to treat FM and ME/CFS on miRNA profiles in an effort to discern between miRNAs potentially linked to disease from those that might be a consequence of drug intake. We have also evaluated miRNA–target gene–drug interactions of differentially expressed (DE) miRNAs in FM and ME/CFS as an approach to determine the ability or predisposition of these patients to respond to common clinical treatments for diseases in general, including diseases other than FM and ME/CFS, which may appear comorbid at some point in FM and ME/CFS patients’ lives.

## 2. Materials and Methods 

### 2.1. Study Search

To locate experimental work aimed at studying miRNA profiles in FM or ME/CFS, a bibliographic search following the Preferred Reporting Items for Systematic Reviews and Meta-Analyses (PRISMA) criteria [58] was performed using Pubmed and Web of Science databases [59,60] up to January 2019. The search terms used in “all fields” included: “fibromyalgia” AND “microRNA” OR “miR” on one search, and “chronic fatigue syndrome” AND “CFS” in combination with “microRNA” OR “miR” in another. The use of the term “myalgic encephalomyelitis” to describe the disease in other searches did not yield any additional experimental publications in the field. The trial Pubmed Labs tool, including article snippets and other improvements was also used in the search [61]. Manual curation to filter out non-experimental or unrelated hits was applied. 

For compounds commonly prescribed to treat symptoms in FM and ME/CFS, a search in the Cochrane library and Pubmed databases [59,62] was performed using as search terms either “fibromyalgia systematic review” AND “drug,” “chronic fatigue syndrome” AND “CFS systematic review” AND “drug,” or “myalgic encephalomyelitis systematic review” AND “drug.” Most recently updated reviews were adopted as reference manuscripts. 

### 2.2. Identification of miRNA–Drug–Disease Interactions 

Features of miRNA and drug understudies, International Union of Pure and Applied Chemistry (IUPAC) names included, were found in miRBase and Drugbank databases, respectively [63,64]. FM and ME/CFS miRNomes were evaluated for miRNA–drug interactions using either SM2miR or Pharmaco-miR web search tools [50,65]. To find potential miRNomes derived from patient polypharmacy, SM2miR output was filtered using as criteria “drugs commonly prescribed to treat FM and ME/CFS symptoms,” as described in the previous Section 2.1. Treatments to disease to which FM and ME/CFS patients may respond differently from non-FM and non-ME/CFS populations were spotted by searching the repoDB database [66] with the Pharmaco-miR drug hits obtained with FM or ME/CFS miRNome searches.

## 3. Results

### 3.1. miRNomes Associating with the Studied Diseases 

#### 3.1.1. miRNomes of FM

By reviewing the literature, as described in Methods, we found five studies reporting differential expression (DE) of particular miRNAs in FM patients with respect to healthy populations using multiplex approaches, either microarrays or RT-qPCR panels (Table 1 and Table S1). One of them measured miRNA levels in cerebrospinal fluid (CSF) [67], while the rest evaluated them in blood fractions [68,69,70,71]—two used white blood cells [69,71] and two analyzed serum [68,70]. 

According to these reports, a total of 85 FM patients and 86 healthy participants were screened for differential miRNA expression, and little coincidence was found (only 9 miRNAs reported by more than one study) (Table 1, miRNAs in bold) even within the same blood fraction type and in spite of using common diagnostic criteria (ACR 1990). Gene Ontology (GO) analysis, however, more commonly showed metabolic and neural pathways associating to DE miRNAs, indicating common cellular pathways affected by different FM miRNomes.

#### 3.1.2. miRNomes of ME/CFS

A similar bibliographic search to the one performed in FM (Section 3.1.1 of this manuscript) yielded, after filtering out unrelated, gene-focused studies, only three studies showing DE of miRNAs in ME/CFS at basal levels, yet, they included a similar total number of patients and controls (83 and 47, respectively) (Table 2) [72,73,74]. It should be noted that an additional multiplex miRNA study evaluating the DE of miRNAs in ME/CFS upon an exercise challenge was excluded on the basis of reporting no basal disease miRNomes [75]. Again, as in FM studies, little overlap of DE miRNAs could be found across ME/CFS studies (only 4 miRNAs were reported by more than one ME/CFS study, bold miRNAs in Table 2). In this case, this could be somehow expected as blood fractions and diagnostic criteria varied across studies. In fact, only the most recent study by Petty et al. included the more restrictive Canadian criteria for patient selection [74]. Nevertheless, once more, a coincidence of mainly affected GO terms was found, indicating major immune defects in ME/CFS through different miRNomes.

Surprisingly as many as 19 miRNAs were found to be commonly reported as DE by FM and ME/CFS studies, the significance of which is unknown at present (miRNAs underlined in Table 1 and Table 2).

### 3.2. Polypharmacy Potentially Impacting miRNA Profiles

As mentioned above, our general aims included determining drug–miRNA and drug–disease interactions in the context of FM, or ME/CFS miRNomes, for the purpose of identifying potential interference of drugs in miRNA profiling, which could bias research outcomes on one hand and, on the other, determine whether disease miRNA profiles could influence drug response in these patients. This section focuses on selecting drugs commonly prescribed to FM and ME/CFS patients to evaluate the effect that polypharmacy might have on miRNomes of these diseases.

#### 3.2.1. Polypharmacy in FM

Based on the recent Cochrane report by Häuser et al. [44], drugs that have been commonly used to treat FM in the clinical practice can be classified into the following six classes: antidepressants, antiepileptics, antipsychotics, cannabinoids, nonsteroidal anti-inflammatory drugs (NSAIDs), and opioids. Rather than analyzing the quality of evidence of clinical trials using these substances, we were interested in assigning the active principle and IUPAC names to the reported compounds, to facilitate our downstream analysis (Table 3). Additional literature supporting the use of compounds for each of the six classes described by Häuser et al. to treat FM patients is provided in Table 3 [76,77,78,79,80,81,82,83,84,85,86,87,88,89,90,91,92,93]. 

#### 3.2.2. Polypharmacy in ME/CFS 

Opposite to FM, no drug-based Cochrane review for the treatment of ME/CFS could be found. The three hits obtained by using the MeSH search terms “chronic fatigue syndrome” were reviews on exercise, CBT (cognitive behaviour therapy) and Chinese herbs [94,95,96]. Therefore, we decided to use the recent reviews by Collatz et al. and Smith et al. as reference papers to evaluate common drug-based ME/CFS therapies [46,47]. Additional bibliography supporting the use of polypharmacy in ME/CFS was also included [46,48,97,98,99,100,101,102,103,104,105]. Similar to what has been described in Section 3.2.1, a documented summary of drugs commonly prescribed to ME/CFS patients that could impact miRNA screenings is shown in Table 4 together with active principles and IUPAC names.

Although possibly not complete, Table 3 and Table 4 include the most representative compounds to treat FM and ME/CFS according to the consulted authors [44,48,76,77,78,79,80,81,82,83,84,85,86,87,88,89,90,91,92,93,97,98,99,100,101,102,103,104,105]. Unexpectedly, a single IUPAC overlap, corresponding to the selective serotonin reuptake inhibitor (SSRI) fluoxetine, was found for drugs commonly prescribed for FM and ME/CFS (in bold in Table 3 and Table 4), indicating little prescription overlap at the IUPAC name level despite both groups of patients presenting common symptomatology. Special attention should be placed to common prescriptions as they may more readily allow for identifying the effects of drugs on miRNA levels over disease-related changes.

### 3.3. miRNA–Drug Interactions in FM and ME/CFS

With the intention to discriminate whether the miRNomes proposed to associate with FM or with ME/CFS are derived from drug intake differences between the patient and control groups, we performed a screen of drugs that could alter any of the miRNAs in these miRNomes using the SM2miR web server [65] and each of the individual DE miRNAs or disease miRNome as the input, as previously detailed in the Methods section.

The SM2miR drug output file (Table S2) was contrasted with the FM and ME/CFS polypharmacy tables (Table 3 and Table 4), and it was found that five of the commonly prescribed drugs for FM or ME/CFS (DHA, fluoxetine, glucocorticoids, morphine, and valproate) are estimated to alter the levels of one or more of the miRNAs found DE in FM or ME/CFS screenings (potential disease-associated miRNomes) and therefore these drugs could constitute confounding variables of the assay (Table 5) [106,107,108,109,110,111]. Overlapping tendencies may suggest that the detected differences between studied groups associate with treatment rather than constituting potential biomarkers of disease, while opposed tendencies might reflect additional factors leading to differential expression other than drug intake, disease status included. Importantly, as summarized in Table 5, the expression of miRNA-27b reported in miRNomes of both FM and ME/CFS in more than one report [67,74] is affected by the only overlapping compound commonly prescribed for treatment of both diseases (fluoxetine), indicating a potential drug–interference effect. Three additional miRNAs reported as miRNomes of ME/CFS by more than one study (miR-26a, miR-126, and miR-191) are also affected by drugs frequently prescribed to ME/CFS patients, so special attention should be paid when interpreting miRNome results including these miRNAs.

It must be pointed out that, in an effort to complete the search as much as possible, the list of DE miRNAs in FM and ME/CFS used in the SM2miR search not only included the miRNAs listed in Table 1 and Table 2 but also those documented in the supplementary tables of the listed literature [67,68,69,70,71,72,73,74].

### 3.4. Drug–Disease Interactions Based on FM and ME/CFS miRNomes

To evaluate potential biased responses of FM and ME/CFS patients to pharmacotherapy in general, due to their DE miRNA profiles, we searched for diseases commonly treated with small-molecule drugs that depend on gene sets linked to FM or ME/CFS miRNomes (miRNA–gene–drug datasets). With this purpose, individual DE miRNAs in FM or ME/CFS were used as input in the Pharmaco-miR web search tool [50]. The output constituted a list of genes whose expression is dependent on FM and ME/CFS DE miRNAs (Table S3) and a third column facilitating small molecule drug associations for these gene lists. Among the 709 small molecules linked to FM miRNome, only 595 appeared registered in the Drugbank database. Out of the 668 small molecules associating with ME/CFS miRNomes, 557 appeared registered in Drugbank [64].

Finally, Drugbank numbers of these small molecules were used as the input to search repoDB, a database of small drugs developed by Brown and Patel to facilitate screenings for drug repositioning [66]. The results (Table S3, miRNome–drug–disease tabs) show 1480 and 1455 diseases treated with small molecules, respectively, associating with FM or ME/CFS miRNomes after filtering out duplications. Out of these diseases potentially impacting individualized medicine programs for FM and ME/CFS patients, more than 30% corresponded to cancer of some type. Within cancer, 13% corresponded to lymphoma, and 14% to lymphoma plus leukemia. This seems to indicate that quite possibly FM and ME/CFS patients may respond differently to treatments for these diseases with respect to non-FM and non-ME/CFS patients, so it is advised that attention be paid to individualized medicine programs for the treatment of these cancers in the case of FM and CFS/ME patients.

## 4. Discussion

This paper is the first to evaluate the relationship between commonly prescribed drugs for FM and ME/CFS and miRNA expression and compares these profiles to FM- and ME/CFS-reported miRNomes in an effort to discern miRNAs presenting differential expression due to medication from differences more likely related to disease. The resources used in this study are limited and therefore it is expected that the evidence presented here will be refined as more data becomes available. The topic is not exclusive to FM and ME/CFS, as it can be extended to any other study evaluating miRNomes associated with disease. However, the fact that FM and ME/CFS patients are usually polymedicated to palliate the multiple symptoms that associate with these illnesses extends this concern to higher levels, particularly demanding careful registry of study participants’ medication, when restrictive medication inclusion criteria is not an option. In this sense, the ME/CFS Common Data Elements initiative [49] has made publicly available medication guidelines and CRFs at the disposition of researchers, which may help standardize medication registry in ME/CFS studies.

Although some researchers have expressed their concern of the impact of drug use by FM and ME/CFS patients on the study of molecular markers and although recent work in the area is already reporting the medication used by participants [71,112], the information of registered drugs is not yet used to evaluate potential interference or bias of results. To elevate biomarker screenings of FM and ME/CFS based on miRNA profiles, complex stratified analysis to filter out potential drug and other confounding variables will be required. The complexity and limitations of this analysis is served by the fact that miRNA expression responds to many cues, such as exercise and diet, hormones, sex, and aging [38,39,40,41,42,43].

A commonly used approach to minimize confounding variables, although not free of certain difficulties for sampling, is to set restrictive inclusion criteria including sex selection, narrow age range, and BMI. This is important in miRNA screenings as these parameters are known to affect miRNA profiles [113]. Additional sampling details such as fasting blood draw and the selection of sedentary healthy controls may improve study outcomes. Some authors have even taken into account time of blood collection to reduce circadian variation [71], but it may not be possible to eliminate polypharmacy, particularly in studies including severely affected FM and ME/CFS patients.

Prescriptions for other common health problems such as diabetes or high cholesterol, diet supplements and some recreational drugs alter the expression of some miRNAs in FM and ME/CFS miRNomes (Table S4) [107,114,115,116,117,118,119,120,121,122,123,124,125]. Hormones and other natural compounds also impact FM and ME/CFS miRNomes (Table S5) [106,126,127,128,129,130,131,132,133,134,135,136], stressing the necessity for researchers to collect complete medical histories of participants to accurately evaluate miRNAs as biomarkers of these diseases.

Though FM and ME/CFS miRNomes relate to disease or derive from chronic polypharmacy use, DE miRs should represent a relevant factor to take into account when treatments for other diseases such as cancer are due. Here, we performed an analysis of the diseases whose treatment response could differ in the context of FM and ME/CFS miRNomes, and found a broad range of them. The major representation of cancer (above 35%) might merely reflect the fact that more studies are registered in the field, biasing databases. Importantly, a relevant number of hits associated with lymphoma, a type of cancer appearing at higher incidence among ME/CFS patients [137], is possibly due to associated immune dysfunctions of this disease.

Personalized medicine programs considering miRNome backgrounds may more adequately select effective treatments with reduced side effects. It is therefore envisioned that future improved therapeutic analysis, including pharmacogenomics and pharmacoepigenomics (precision medicine programs), will rely on complex software tools fed with large datasets. Further miRNA profiling studies including a larger number of samples are required to build on the scarce available FM and ME/CFS miRNome data. Since technical variability in miRNA qPCR replicates has been documented, with TaqMan overweighing qScript PCR [138], future studies should include repeated independent measures or either use alternative enzyme-free approaches such as NanoString [139].

In general, we have evaluated the effects of polypharmacy and miRNomes at individual levels, meaning that the information obtained here corresponds to the effects of a single drug on DE miRNAs or the impact of an individual miRNA on drug performance, but the effects of combined therapies on miRNA profiles or sets of DE miRNAs on drug response may not replicate or be additive of single events, highlighting the limitation of our study. In addition, most molecular data come from analysis of blood or other body fluid samples and more sparingly from non-cancerous solid tissues, limiting the validity of our results, as miRNA profiles are known to be tissue-restricted [140]. Drug assays are performed in either animal models or tumor cell lines leading to results that may not replicate in other systems, especially since many miRNAs are primate or human-specific [63,141].

In summary, as larger data sets become available to nurture databanks, biomarker discovery will be facilitated and personalized medicine programs will be refined, upgrading current diagnostic tools and clinical treatments. Drug–transcriptome interactions are key factors in either context, particularly in diseases subject to polypharmacy such as FM and ME/CFS.

## 5. Conclusions

The analysis presented here seem to support a potential impact of FM and ME/CFS polypharmacy in the discovery of miRNomes associating with these diseases. Based on this possibility, caution is advised when designing studies aimed at determining DE miRNAs linked to these diseases, including complete drug registry to permit stratified analysis.

FM and ME/CFS miRNomes may predispose patients to respond differently to a large variety of drug-based treatments, including those used for a large number of cancers, highlighting the importance of considering this epigenomic bias in refined personalized programs towards improving a patient’s response to clinical treatments while minimizing toxicity. It is estimated that more sophisticated informatic tools will help with these predictions, but the paucity of molecular studies in FM and ME/CFS currently limits their development.

The results presented here are not definitive at this stage, but their observations should stimulate additional studies to further explore miRNA–drug interactions in the context of FM and ME/CFS.

## Figures and Tables

**Table 1:** Summary of studies evaluating fibromyalgia (FM) miRNomes by multiplex approaches.

Source of RNA	Diagnostic Criteria /Clinical Parameters	Cohorts	Technical Approach	Over-Expressed microRNAs	Under-Expressed microRNAs	RT-qPCR Validated miRNAs	GO Terms Mainly Affected	References
Cerebrospinal fluid (CSF)	ACR 1990, FIQ & MFI-20 *	10 FM 8 HC	microRNA Ready-to-Use PCR microchip (Exiqon, Denmark Cat No 203608)		**miR-21-5p**, **miR-145-5p**, miR-29a-3p, miR-99b-5p, miR-125b-5p, **miR-23a-3p**, miR-23b-3p, miR-195-5p, **miR-223-3p**	N/A	Glial and neuronal response, insulin-like growth factor pathway, Alzheimer’s and Parkinson’s, autoimmunity and energy metabolism	Bjersing et al., 2013 [67]
Serum	ACR 1990, FIQ & MFI-20 *	20 FM 20 HC	microRNA Ready-to-Use PCR microchip (Exiqon, Denmark Cat No 203608)	**miR-320a**	**miR-103a-3p**, **miR-107**, let-7a-5p, mir-30b-5p, **miR-151a-5p**, miR-142-3p, miR-374b-5p.	N/A	Neuronal regeneration, opioid tolerance, dopamine neurotransmitter receptor activity, cell division, stress response, energy metabolism, lipid metabolism, Alzheimer’s	Bjersing et al., 2014 [68]
PBMCs	ACR 1990, FIQ & MFI-20 *	11 FM 10 HC	3D-Gene Human miRNA Oligo chips (version 16.0; Toray Industries)		**miR-223-3p**, miR-451a, miR-338-3p, miR-143-3p, **miR-145-5p**, **miR-21-5p**	miR-223-3p, miR-451a, miR-338-3p, miR-143-3p, miR-145-5p	Nervous system, inflammation, diabetes, major depressive disorder	Cerdá-Olmedo et al., 2015 [69]
Serum	ACR 1990/2010, FIQ, FAS, HAQ & ZSAS/ZSDS *	14 FM 14 HC	Serum/Plasma Focus miRNA PCR Panel I+II (96-wells Exiqon)	Pooled Sera: miR-10a-5p, **miR-320b**, miR-424-5p	Pooled Sera: miR-20a-3p, miR-139-5p **Individual Sera**: **miR-23a-3p**, miR-1, miR-133a, miR-346, miR-139-5p, **miR-320b**	N/A	Brain development, immune response, osteogenesis, myoblast differentiation, autism, epilepsy, cellular proliferation and differentiation, muscular atrophy, complex regional pain syndrome, among others	Masotti et al., 2016 [70]
White blood cell (WBC)	ACR 1990, FIQ, NPSI-G, GCPS & ADS *	30 FM 34 HC	miRCURY LNA miRNA array (Exiqon, Vedbaek, version 19.0, with 2042 analyzed microRNAs)	miR-136-5p, miR-4306, miR-744-5p, miR-4301, miR-151a-3p, miR-584-5p, miR-4288, miR-221-3p, **miR-151a-5p**, miR-199a-5p, miR-126-3p, miR-126-5p, miR-130a-3p, miR-146a-5p, miR-125a-5p, miR-4429, **miR-320b**, miR-320a, miR-320c, miR-17-3p, miR-423-3p, miR-425-5p, miR-4291, miR-652-3p, miR-103b-3p, miR-199a-3p, miR-335-5p, miR-331-3p, miR-339-5p, miR-92a-3p, let-7b-5p, miR-222-3p, miR-33a, let-7i-5p, miR-185-5p, miR-22-3p, miR-148b-3p, **miR-103a-3p**, let-7d-5p, miR-4289, **miR-107**, miR-30d-5p, miR-301a-3p, miR-374c-5p, miR-17-5p, miR-18b-5p, miR-1	miR-4639-3p, miR-3685, miR-943, miR-877-3p	miR-199a, miR-151, miR-103, Let-7d, miR-146a	Cell proliferation, differentiation, brain development, opioid tolerance	Leinders et al., 2016 [71]

* ACR: American College of Rheumatology 1990/2010 criteria; FIQ; Fibromyalgia Impact Questionnaire; MFI-20: Multidimensional Fatigue Inventory; FAS: Fibromyalgia Assessment Status; HAQ: Health Assessment Questionnaire; ZSAS, ZSDS: Zung Self-Rating Anxiety and Zung Self-Rating Depression Scale; NPSI-G: Neuropathic Pain Symptom Inventory; GCPS: Graded Chronic Pain Scale; ADS: Allgemeine Depressions-Skala. Bolded miRs correspond to miRs differentially expressed (DE) according to more than one FM study. Underlined miRs correspond to miRs DE in FM and myalgic encephalomyelitis/chronic fatigue syndrome (ME/CFS) studies.

**Table 2:** Summary of studies evaluating ME/CFS miRNomes by multiplex approaches.

Source of RNA	Diagnostic Criteria	Cohorts	Technical Approach	Over-Expressed microRNAs	Under-Expressed microRNAs	RT-qPCR Validated microRNAs	GO Terms Mainly Affected	References
NK & CD8+ cells	Fukuda	28 ME/CFS 28 HC	Analyzed by RT-qPCR 19 microRNAs: miR-10a miR-16, miR-15b, miR-107, miR-128b, miR-146a, miR-191, miR-21, miR-223, miR-17-5p, miR-150, miR-103, miR-106b, miR-126, miR-142-3p, miR-146-5p, miR-152, miR-181, let-7a.		NK: miR-10a, miR-146a, **miR-191**, miR-223, miR-17-5p, miR-21, miR-106, miR-152, **miR-103** CD8^+^: miR-21	N/A	Cytotoxicity of NK and CD8^+^ cells, cytokine expression, cell proliferation, apoptosis, development and differentiation of effector CD8^+^	Brenu et al., 2012 [72]
Plasma	Fukuda	20 ME/CFS 20 HC	MicroRNA profiling by HiSeq2000 sequencing (Illumina HiSeq2000)	miR-548j, miR-548ax, miR-127-3p, miR-381-3p, **miR-331-3p**, miR-136-3p, miR-142-5p, miR-493-5p, miR-143-3p, miR-370, miR-4532	**miR-126**, miR-450b-5p, miR-641, miR-26a-1-3p, miR-3065-3p, miR-5187-3p, miR-16-2-3p, let-7g-3p	miR-127-3p, miR-142-5p, miR-143-3p	Autoimmunity, T cell development, cytokine production, inflammatory responses, apoptosis	Brenu et al., 2014 [73]
PBMCs	Fukuda & Canadian	35 ME/CFS 50 HC	Ambion Bioarray microarrays (version 1 targeting 385 miRNA sequences)	let-7b, **miR-103**, **miR-126**, miR-145, miR-151, miR-181a, miR-185, **miR-191**, miR-197, miR-199a, miR-19b, miR-210, miR-22-5p, miR-24, miR-27a, miR-27b, miR-30c, miR-30d, miR-320, miR-324-3p, miR-324-5p, miR-326, miR-330, **miR-331-3p**, miR-339, miR-422b, miR-423, miR-92, miR-99b		miR-99b, miR-30c, miR-126, miR-330-3p	Angiogenesis, invasion, migration and proliferation in dendritic cells, proliferative, cytotoxic and cytokine effector function in NK cells	Petty, et al., 2016 [74]

Bolded miRs correspond to miRs DE according to more than one ME/CFS study. Underlined miRs correspond to miRs DE in FM and ME/CFS studies. This table has been adapted from Almenar-Perez, E.; Ovejero, T.; Sánchez-Fito, T.; Espejo, J.A.; Nathanson, L.; Oltra, E. Epigenetic components of Myalgic Encephalomyelitis/Chronic Fatigue Syndrome (ME/CFS) uncover potential transposable element activation (Clin Ther, accepted, special issue: “Immunology Specialty Update on CFS/ME.”, Elsevier 2019).

**Table 3:** Classification of drugs commonly prescribed to FM patients.

Family	Subfamily	Active Principle	IUPAC Name	Reference
Antidepressants	Serotonin-Norepinephrine reuptake inhibitors (SNRIs)	Milnacipran	(±)-(1*R*,2*S*)-rel-2-(Aminomethyl)-*N*,*N*-diethyl-1-phenylcyclopropane-1-carboxamide	Cording M et al., 2015 [76]
		Duloxetine	(+)-(*S*)-*N*-Methyl-3-(naphthalen-1-yloxy)-3-(thiophen-2-yl)propan-1-amina	Lunn MP et al., 2014 [77]
	Selective serotonin reuptake inhibitors (SSRIs)	Citalopram	(*RS*)-1-[3-(dimethylamino) propyl]-1-(4-fluorophenyl)-1,3-dihydroisobenzofuran-5-carbonitrile	Walitt B et al., 2015 [78]
		**Fluoxetine**	**(*RS*)-*N*-Methyl-3-phenyl-3-(4-trifluoromethylphenoxy) propylamine**	
		Paroxetine	(3*S*, 4*R*)-3-[(1,3-Benzodioxol-5-yl oxy) methyl]-4-(4-fluorophenyl) piperidine	
		Tryptophan	2-amino-3-(1H-indol-3-yl) propanoic acid	
		Escitalopram	(*S*)-1-[3-(Dimethylamino)propyl]-1-(4-fluorophenyl)-1,3-dihydroisobenzofuran-5-carbonitrile	Riera R, 2015 [79]
		Sertraline	(1*S*,4*S*)-4-(3,4-dichlorophenyl)-*N*-methyl-1,2,3,4-tetrahydronaphthalen-1-amine	
	Tricyclic antidepressants	Amitriptyline	8-methyl-2,3,3a,4,5,6-hexahydro-1*H*-pyrazino[3,2,1-jk]carbazole	Moore RA et al.,2015 [80]
	Monoamine oxidase inhibitors (MAOIs)	Pirlindole	8-methyl-2,3,3a,4,5,6-hexahydro-1*H*-pyrazino[3,2,1-jk]carbazole	Tort S et al., 2012 [81]
		Moclobemide	4-chloro-*N*-(2-morpholin-4-ylethyl) benzamide	
		Mirtazapine	(*RS*)-1,2,3,4,10,14b-Hexahydro-2-methylpyrazino[2,1-*a*]pyrido[2,3-c][2]benzazepine	Welsch P et al., 2018 [82]
Antiepileptics	1st Generation	Phenytoin	5,5-diphenylimidazolidine-2,4-dione	Birse F et al., 2012 [83]
	2nd Generation	Valproic acid (Sodium valproate)	2-propylpentanoic acid	Gill D et al., 2011 [84]
		Clonazepam	5-(2-Chlorophenyl)-7-nitro-1,3-dihydro-1,4-benzodiazepin-2-one	Corrigan R et al., 2012 [85]
	3rd Generation	Pregabalin	(*S*)-3-(amynomethyl)-5-methylhexanoic acid	Derry S et al., 2016 [86]
		Gabapentin	2-[1-(amynomethyl)cyclohexyl]ethanoic acid	Wiffen PJ et al., 2017 [87]
		Lacosamide	N2-acetyl-*N*-benzyl-d-homoserinamide	Hearn L et al., 2016 [88]
		Topiramate	2,3: 4,5-Bis-O-(1-methylethylidene)-beta-d-fructopyranose sulfamate	Wiffen PJ et al., 2013 [89]
Antipsychotics	Atypical	Quetiapine	2-(2-(4-dibenzo [b,f] [1,4] thiazepine-11-yl-1-piperazinyl) ethoxy) ethanol	Walitt B et al., 2016 (Jun) [90]
Cannabinoids	Synthetic	Nabilone	(6a*R*,10a*R*)-rel-1-Hydroxy-6,6-dimethyl-3-(2-methyl-2-octanyl)-6,6a,7,8,10,10a-hexahydro-9*H*-benzo[c]chromen-9-one	Walitt B et al., 2016 (Jul) [91]
**Nonsteroidal anti-inflammatory drugs (NSAIDs)**	Selective inhibitor of Cyclooxygenase 2 (COX-2)	Etoricoxib	5-cloro-6′-metil-3-[4-(metilsulfonil)fenil]-2,3′-bipiridine	Derry S et al., 2017 [92]
	Inhibitor of prostaglandin synthesis	Ibuprofen	(*RS*)-2-(4-(2-Methylpropyl)phenyl)propanoic acid	
		Naproxen	(+)-(*S*)-2-(6-Methoxynaphthalen-2-yl)propanoic acid	
	Inhibitor of Cicloxygenase (COX-1 and COX-2)	Tenoxicam	(3E)-3-[hydroxy(pyridin-2-ylamino)methylene]-2-methyl-2,3-dihydro-4H-thieno[2,3-e] [1,2]thiazin-4-one 1,1-dioxide	
Opioids	Semi synthetic	Oxycodone	(5*R*,9*R*,13*S*,14*S*)-4,5-α-epoxy-14-hydroxy-3-methoxy-17-methyl-morphinan-6-one	Gaskell H et al., 2016 [93]

Drugs commonly prescribed to both FM and ME/CFS patients are bolded.

**Table 4:** Classification of drugs commonly prescribed to ME/CFS patients.

Family	Subfamily	Active Principle	IUPAC Name	Reference
**Anticonvulsants**	3rd Generation	Gabapentin	2-[1-(amynomethyl)cyclohexyl]ethanoic acid	Castro-Marrero J et al., 2017 [48]
		Pregabalin	(S)-3-(amynomethyl)-5-methylhexanoic acid	
**Antidepressants**	Selective serotonin reuptake inhibitors (SSRIs)	Nafazodone	2-[3-[4-(3-chlorophenyl)piperazin-1-yl]propyl]-5-ethyl-4-(2-phenoxyethyl)-1,2,4-triazol-3-one	Collatz A et al., 2016 [46]
		Bupropion	(RS)-2-(tert-Butylamino)-1-(3-chlorophenyl)propan-1-one	Castro-Marrero J et al., 2017 [48]
		Citalopram	((RS)-1-[3-(Dimethylamino)propyl]-1-(4-fluorophenyl)-1,3-dihydroisobenzofuran-5-carbonitrile	
		Escitalopram	((S)-1-[3-(Dimethylamino)propyl]-1-(4-fluorophenyl)-1,3-dihydroisobenzofuran-5-carbonitrile	
		**Fluoxetine**	**(RS)-N-Methyl-3-phenyl-3-(4-trifluoromethylphenoxy) propylamine**	
		Sertraline	(1S,4S)-4-(3,4-dichlorophenyl)-N-methyl-1,2,3,4-tetrahydronaphthalen-1-amine	
		Paroxetine	(3S, 4R)-3-[(1,3-Benzodioxol-5-yl oxy) methyl]-4-(4-fluorophenyl) piperidine	
	Serotonin–norepinephrine reuptake inhibitors (SNRIs)	Methylphenidate	Methyl phenyl(piperidin-2-yl)acetate	Blockmans D and Persoons P, 2016 [97]; Castro-Marrero J et al., 2017 [48]
		Duloxetine	(+)-(S)-N-Methyl-3-(naphthalen-1-yloxy)-3-(thiophen-2-yl)propan-1-amine	Castro-Marrero J et al., 2017 [48]
		Venlafaxine	(RS)-1-[2-dimethylamino-1-(4-methoxyphenyl)-ethyl]cyclohexanol	
	Tricyclic antidepressants	Amitriptyline	3-(10,11-dihydro-5H-dibenzo [a,d] cycloheptene-5-ylidene)-N, N-dimethyl-1-propanamine	Castro-Marrero J et al., 2017 [48]
		Clomipramine	3-(2-chloro-5,6-dihydrobenzo[b][1]benzazepin-11-yl)-N,N-dimethylpropan-1-amine	
		Desipramine	3-(5,6-dihydrobenzo[b][1]benzazepin-11-yl)-N-methylpropan-1-amine	
		Doxepin	(3E)-3-(6H-benzo[c][1]benzoxepin-11-ylidene)-N,N-dimethylpropan-1-amine	
		Imipramine	3-(5,6-dihydrobenzo[b][1]benzazepin-11-yl)-N,N-dimethylpropan-1-amine	
		Nortriptyline	3-(5,6-dihydrodibenzo[2,1-b:2′,1′-f][7]annulen-11-ylidene)-N-methylpropan-1-amine	
	Monoamine oxidase inhibitors (MAOIs)	Moclobemide	4-chloro-N-(2-morpholin-4-ylethyl)benzamide	Collatz A et al., 2016 [46]; Castro-Marrero J et al., 2017 [48]
		Phenelzine	2-phenylethylhydrazine	
		Selegiline	(R)-N-methyl-N-(1-pheny lpropan-2-yl)prop-1-yn-3-amine	Castro-Marrero J et al., 2017 [48]
	Noradrenergic and specific serotonin antagonist (NaSSAs)	Mirtazapine	(RS)-1,2,3,4,10,14b-Hexahydro-2-methylpyrazino[2,1-a]pyrido[2,3-c][2]benzazepine	Castro-Marrero J et al., 2017 [48]
	Monoaminergic stabilizer	(–)-OSU-6162	(3S)-3-[3-(methylsulfonyl)phenyl]-1-propylpiperidine	Nilsson MKL et al., 2017 [98]
**Antihypertensive**	Stimulant to α2-Receptors	Clonidine hydrochloride	N-(2,6-dichlorophenyl)-4,5-dihydro-1H-imidazol-2-amine;hydrochloride	Collatz A et al., 2016 [46]
	Angiotensin II receptor agonist	Olmesartan medoxomil	(5-metil-2-oxo-2H-1,3-dioxol-4-il)metil 4-(2-hidroxipropan-2-il)-2-propil-1-({4-[2-(2H-1,2,3,4-tetrazol-5-il)fenil]fenil}metil)-1H-imidazole-5-carboxilato	Proal AD et al., 2013 [99]
**Antioxidant**	Fatty acid oxidant	l-Carnitine	3-Hydroxy-4-(trimethylazaniumyl)butanoate	Plioplys AV and Plioplys S., 1997 [100]
	Ubiquinone	CoQ10	[(2E,6E,10E,14E,18E,22E,26E,30E,34E)-3,7,11,15,19,23,27,31,35,39-Decamethyltetraconta-2,6,10,14,18,22,26,30,34,38-decaenyl]-5,6-dimethoxy-3-methylcyclohexa-2,5-diene-1,4-dione	Castro-Marrero J et al., 2015 [101]
	Re-Dox Agent	NADH	Nicotine adenine dinucleotide	
	Omega-3 fatty acid	α-lipoic acid	(R)-5-(1,2-dithiolan-3-yl)pentanoic acid	Castro-Marrero J et al., 2017 [48]
		Docosahexaenoic acid(DHA)	(4Z,7Z,10Z,13Z,16Z,19Z)-docosa-4,7,10,13,16,19-hexaenoic acid	
	Vitamins	Vitamin C	(2R)-2-[(1S)-1,2-dihydroxyethyl]-3,4-dihydroxy-2H-furan-5-one	
		Folate (Vitamin B9)	(2S)-2-[[4-[(2-Amino-4-oxo-1H-pteridin-6-yl)methylamino]benzoyl]amino]pentanedioic acid	
		Hydroxycobalamin Vitamin B12)	Coα-[α-(5,6-dimethylbenzimidazolyl)]-Coβ-hydroxocobamide	
Antiviral	Blocking adhesion and viral penetration	Amantadine	1-amino-adamantane	Plioplys AV and Plioplys S., 1997 [100]
	Acid nucleics analogs	Valganciclovir	[2-[(2-amino-6-oxo-3H-purin-9-yl)methoxy]-3-hydroxypropyl] (2S)-2-amino-3-methylbutanoate	Collatz A et al., 2016 [46]; Castro-Marrero J et al., 2017 [48]
		Acyclovir	2-amino-9-(2-hydroxyethoxymethyl)-3H-purin-6-one	Castro-Marrero J et al., 2017 [48]
		Valacyclovir	2-[(2-amino-6-oxo-3H-purin-9-yl)methoxy]ethyl (2S)-2-amino-3-methylbutanoate	
Corticoids	Glucocoticoid	Hydrocortisone	(11β)-11,17,21-trihydroxypregn-4-ene-3,20-dione	Blockmans D et al., 2003 [102]; Collatz A et al., 2016 [46]
		Fludrocortisone	(8S,9R,10S,11S,13S,14S,17R)-9-fluoro-11,17-dihydroxy-17-(2-hydroxyacetyl)-10,13-dimethyl-1,2,6,7,8,11,12,14,15,16-decahydrocyclopenta[a]phenanthren-3-one	Blockmans D et al., 2003 [102]
**Nonsteroidal Anti-Inflammatory Drugs (NSAIDs)**	Inhibitor of prostaglandin synthesis	Ibuprofen	(RS)-2-(4-(2-Methylpropyl)phenyl)propanoic acid	Castro-Marrero J et al., 2017 [48]
		Naproxen	(+)-(S)-2-(6-Methoxynaphthalen-2-yl)propanoic acid	
Others	Immunomodulatory double stranded RNA	Rintatolimod	5′-Inosinic acid, homopolymer, complex with 5′-cytidylic acid polymer with 5′-uridylic acid (1:1)	Strayer DR et al., 2012 [103]
	Anti-neoplastic	Sodium dichloroacetate	Dichloroacetic acid	Comhaire F., 2018 [104]
	Ig gamma-1 chain C region	Rituximab	Lithium;4-[2-(diethylamino)ethylcarbamoyl]-2-iodobenzoate	Collatz A et al., 2016 [46]
	Proliferation inductor from B cells	Intravenous immunoglobulin (Immunoglobulin G)	(2S)-2-[[(2S)-1-[(2S)-6-amino-2-[[(2S,3R)-2-[[(2S)-6-amino-2-[[(2S)-2-[[(2S)-4-amino-2-[[(2S)-2-amino-3-(1H-indol-3-yl)propanoyl]amino]-4-oxobutanoyl]amino]propanoyl]amino]hexanoyl]amino]-3-hydroxybutanoyl]amino]hexanoyl]pyrrolidine-2-carbonyl]amino]-5-(diaminomethylideneamino)pentanoic acid	
	Hormone	Growth hormone (Somatotropin)	191 amino acid peptide (IUPAC name N/A)	
	Wakefulness-promoting	Modafinil	2-[(diphenylmethyl)sulfinul]acetamide	
	Peripherally-selective antihistamine	Terfenadine	1-(4-tert-butylphenyl)-4-[4-[hydroxy(diphenyl)methyl]piperidin-1-yl]butan-1-ol	
	Precursor of Creatine	Guanidinoacetic acid (Glycocyamine)	2-(diaminomethylideneamino)acetic acid	Ostojic SM et al., 2016 [105]
Pain	Opiate	Codeine	(5α,6α)-7,8-didehydro-4,5-epoxy-3-methoxy-17-methylmorphinan-6-ol	Castro-Marrero J et al., 2017 [48]
		Morphine	(4R,4aR,7S,7aR,12bS)-3-Methyl-2,3,4,4a,7,7a-hexahydro-1H-4,12-methanobenzofuro[3,2-e]isoquinoline-7,9-diol	
	Opiod	Tramadol	(±)-cis-2-[(dimetilamino)metil]-1-(3-metoxifenil) ciclohexanol hidrocloruro	
Psycho-pharmaceutical	Benzodiazepine	Galantamine hidrobromide	(4aS,6R,8aS)-5,6,9,10,11,12-Hexahydro-3-methoxy-11-methyl-4aH-[1]benzofuro[3a,3,2-ef][2]benzazepin-6-ol	Collatz A et al., 2016 [46]
	Psychostimulant	Dextroamphetamine	(2S)-1-phenylpropan-2-amine	

Drugs commonly prescribed to both FM and ME/CFS patients are bolded.

**Table 5:** Effect of FM and ME/CFS polypharmacy on miRNomes associated with disease.

Prescribed Drugs	miR Affected	Disease	miR Levels in Patients	Treatment Effect	Reference
Docosahexaenoic acid (DHA)	miR-30c	ME/CFS	↑ (PBMCs) [74]	Upregulated	Gil-Zamorano J et al., 2014 [106]
	miR-143-3p	ME/CFS	↑ (Plasma) [73]	Upregulated	
	miR-181a-5p	ME/CFS	↑ (PBMCs) [74]	Upregulated	
	miR-330	ME/CFS	↑ (PBMCs) [74]	Upregulated	
Fluoxetine	miR-27b	FM	↓ (CSF) [67]	Upregulated	Rodrigues AC et al., 2011 [107]
		ME/CFS	↑ (PBMCs) [74]		
Glucocorticoid	miR-16	ME/CFS	↓ (Plasma) [73]	Upregulated	Rainer J et al., 2009 [108]
	miR-19b	ME/CFS	↑ (PBMCs) [74]	Upregulated	
	miR-181a	ME/CFS	↑ (PBMCs) [74]	Upregulated	Rainer J et al., 2009 [108]; Lu S et al., 2012 [109]
	miR-223	ME/CFS	↓ (NK cells) [72]	Upregulated	
	miR-21	ME/CFS	↓ (NK cells) [72]	Upregulated	Lu S et al., 2012 [109]
	miR-10a	ME/CFS	↓ (NK cells) [72]	Upregulated	
	miR-27a	ME/CFS	↑ (PBMCs) [74]	Upregulated	
	miR-99b	ME/CFS	↑ (PBMCs) [74]	Upregulated	
	**miR-126**	ME/CFS	↓ (Plasma) [73]	Upregulated	
			↑ (PBMCs) [74]		
	miR-145	ME/CFS	↑ (PBMCs) [74]	Upregulated	
	miR-146a	ME/CFS	↓ (NK cells) [72]	Upregulated	
	miR-324-5p	ME/CFS	↑ (PBMCs) [74]	Upregulated	
	miR-339-3p	ME/CFS	↑ (PBMCs) [74]	Upregulated	
Morphine	miR-16	ME/CFS	↓ (Plasma) [73]	Upregulated	Dave R.S & Khalili K., 2010 [110]
	miR-24	ME/CFS	↑ (PBMCs) [74]	Upregulated	
	miR-30c	ME/CFS	↑ (PBMCs) [74]	Upregulated	
	miR-146a	ME/CFS	↓ (NK cells) [72]	Upregulated	
	miR-21	ME/CFS	↓ (NK cells) [72]	Downregulated	
	**miR-26a**	ME/CFS	↓ (NK cells) [72]	Downregulated	
			↑ (PBMCs) [74]		
	miR-99b	ME/CFS	↑ (PBMCs) [74]	Downregulated	
	**miR-191**	ME/CFS	↓ (NK cells) [72]	Downregulated	
			↑ (PBMCs) [74]		
	miR-320a	ME/CFS	↑ (PBMCs) [74]	Downregulated	
	miR-320c	ME/CFS	↑ (PBMCs) [74]	Downregulated	
	miR-423-5p	ME/CFS	↑ (PBMCs) [74]	Downregulated	
Valproate	miR-21	FM	↓ (PBMCs) [69]	Upregulated	Fayyad-Kazan H et al., 2010 [111]
	miR-125a	FM	↑ (WBC^*^) [71]	Downregulated	

* WBC: white blood cells. Bolded miRs correspond to miRs DE according to more than one ME/CFS study. Underlined miRs correspond to mi Rs DE in FM and ME/CFS studies.

## Supplementary Materials

The following are available online at https://www.mdpi.com/1999-4923/11/3/126/s1. Table S1: PRISMA based search of FM and ME/CFS miRNA profiling studies; Table S2: FM and ME/CFS miRNomes SM2miR-based drug search; Table S3: FM and ME/CFS miRNomes Pharmaco-miR-based gene-drug associations and repoDB-drug-disease screening; Table S4: Effect of additional drugs, supplements and recreational drugs on FM and ME/CFS miRNomes and Table S5: Effect of hormones and other natural compounds on FM and ME/CFS miRNomes.

## Abbreviations

Fibromyalgia (FM); myalgic encephalomyelitis/chronic fatigue syndrome (ME/CFS); microRNAs (miRs); differentially expressed (DE); American College of Rheumatology (ACR); gene ontology (GO); Preferred Reporting Items for Systematic Reviews and Meta-Analyses criteria (PRISMA); International Classification of Diseases (ICD); non-steroidal anti-inflammatory drugs (NSAIDs); cognitive behaviour therapy (CBT); International Union of Pure and Applied Chemistry (IUPAC); concept unique identifiers (CUIs); national clinical trial (NCT); Common Data Elements (CDE); case report forms (CRFs); National Institute of Health (NIH); absorption, distribution, metabolism, and excretion (ADME); selective serotonin reuptake inhibitor (SSRI); serotonin-norepinephrine reuptake inhibitors (SNRIs); monoamine oxidase inhibitors (MAOIs).
