# Supplementary material for: Impact of Polypharmacy on Candidate Biomarker miRNomes for the Diagnosis of Fibromyalgia and Myalgic Encephalomyelitis/Chronic Fatigue Syndrome: Striking Back on Treatments
Source: Pharmaceutics. 2019 Mar 18;11(3):126. doi: 10.3390/pharmaceutics11030126 (PMC6471415; doi:10.3390/pharmaceutics11030126)
Supplement: Supplementary file 1 [file pharmaceutics-11-00126-s001.zip › Table S4.pdf]

Other Drugs	miR affected	Disease	miR levels in patients	Treatment effect	Reference
**Atorvastatin**	miR-182	FM	↓**(CSF*)** [67]	Up-regulated	Peng X *et al.*, 2013[116]
	miR-221	FM	↑**(WBC**)** [71]	Down-regulated	Minami Y *et al.*, 2009[117]
	**miR-222**	FM	↓**(CSF*)** [67] / ↑**(WBC**)** [71]	Down-regulated	
**Celecoxib**	miR-26b	FM	↓**(CSF*)** [67]	Up-regulated	Saito Y *et al.*, 2013[118]
	miR-195	FM	↓**(CSF*)** [67]	Up-regulated	
	miR-29c	FM	↓**(CSF*)** [67]	Down-regulated	
**Isoflavone**	miR-29a	FM	↓**(CSF*)** [67]	Up-regulated	Li Y *et al.*, 2012 (Aug)[119]
	**miR-92a**	FM	↓**(CSF*)** [67] ↑**(WBC**)** [71]	Down-regulated	Li Y *et al.*, 2012 (Mar)[120]
		ME/CFS	↑**(PBMCs)** [74]		
**Metformin**	let-7a	FM	↓**(Serum)** [68]	Up-regulated	Bao B *et al.*, 2012[121]
	miR-101	FM	↓**(CSF*)** [67]	Up-regulated	
	let-7b	FM	↓**(CSF*)** [67] ↑**(WBC**)** [71]	Up-regulated	
		ME/CFS	↑**(PBMCs)** [74]		
	miR-26a	FM	↓**(CSF*)** [67]	Up-regulated	
		ME/CFS	↓**(Plasma)** [73]		
**Cocaine**	miR-27b	FM	↓**(CSF*)** [67]	Up-regulated	Rodrigues AC *et al.*, 2011[109]
		ME/CFS	↑**(PBMCs)** [74]		
	miR-181a	FM	↓**(CSF*)** [67]	Up-regulated	Chandrasekar V & Dreyer JL., 2009[122]
		ME/CFS	↑**(PBMCs)** [74]		
	let-7d	FM	↑**(WBC**)** [71]	Down-regulated	
	**miR-20a**	FM	↓**(CSF*)** [67] / ↓**(Serum)** [70]	Down-regulated	Mantri CK *et al.*, 2014[123]
	miR-125b	FM	↓**(CSF*)** [67]	Down-regulated	
**Ethanol**	miR-27a*	FM	↓**(CSF*)** [67]	Up-regulated	Guo Y *et al.*, 2012[124]
		ME/CFS	↑**(PBMCs)** [74]		
	miR-152	FM	↓**(CSF*)** [67]	Up-regulated	
		ME/CFS	↓**(NK cells)** [72]		
	miR-24-2*	ME/CFS	↑**(PBMCs)** [74]	Up-regulated	
	miR-199a-3p	ME/CFS	↑**(PBMCs)** [74]	Up-regulated	
	miR-182	FM	↓**(CSF*)** [67]	Down-regulated	
**Methamphetamine**	miR-125b	FM	↓**(CSF*)** [67]	Up-regulated	Mantri CK *et al.*, 2014[123]
	miR-150	FM	↓**(CSF*)** [67]	Up-regulated	
	miR-223	FM	↓**(PBMCs)** [69]	Up-regulated	
		ME/CFS	↓**(CSF*)** [67]		
**Nicotine**	miR-16	FM	↓**(CSF*)** [67]	Up-regulated	Shin VY *et al.*, 2011[125]
		ME/CFS	↓**(Plasma)** [73]		
	**miR-21**	FM	↓**(CSF*)** [67] ↓**(PBMCs)** [69]	Up-regulated	Shin VY *et al.*, 2011[125]; Zhang Y *et al.*, 2014[126]
		ME/CFS	↓**(NK cells/ CD8+)** [72]		
	miR-146a	FM	↑**(WBC**)** [71]	Down-regulated	Maccani AM *et al.*, 2010[127]
		ME/CFS	↓**(NK cells)** [72]		

Bolded miRs correspond to miRs DE according to more than one FM or more than one ME/CFS study. Underlined miRs correspond to miRs DE in FM and ME/CFS studies.
