# Supplementary material for: Impact of Polypharmacy on Candidate Biomarker miRNomes for the Diagnosis of Fibromyalgia and Myalgic Encephalomyelitis/Chronic Fatigue Syndrome: Striking Back on Treatments
Source: Pharmaceutics. 2019 Mar 18;11(3):126. doi: 10.3390/pharmaceutics11030126 (PMC6471415; doi:10.3390/pharmaceutics11030126)
Supplement: Supplementary file 1 [file pharmaceutics-11-00126-s001.zip › Table S5.pdf]

Natural molecules	miR affected	Disease	miR levels in patients	Treatment effect	Reference
**17beta-estradiol (E2)**	let-7a	FM	↓(Serum) [68]	Up-regulated	Bhat-Nakshatri P *et al*., 2009[128]
	let-7d	FM	↑(WBC**) [71]	Up-regulated	
	let-7i	FM	↑(WBC**) [71]	Up-regulated	
	**miR-23a**	FM	↓(CSF)[67]<br>↓(Serum)[70]	Up-regulated	
	**miR-30b**	FM	↓(CSF) [67]<br>↓(Serum) [68]	Up-regulated	
	**miR-107**	FM	↓(Serum) [68]<br>↑(WBC**) [71]	Up-regulated	
	**miR-424**	FM	↓(CSF) [67]<br>↑(Serum) [70]	Up-regulated	
	**let-7b**	FM	↓(CSF*) [67]<br>↑(WBC**) [71]	Up-regulated	
		ME/CFS	↑(PBMCs) [74]		
	miR-17-5p	FM	↑(WBC**) [71]	Up-regulated	
		ME/CFS	↓(NK cells) [72]		
	**miR-30c**	FM	↓(CSF*) [67]	Up-regulated	
		ME/CFS	↑(PBMCs) [74]		
	**miR-1**	FM	↓(Serum) [70]<br>↑(WBC**) [71]	Down-regulated	
	miR-526b	FM	↑(CSF*) [67]	Down-regulated	
	miR-27a	FM	↓(CSF*) [67]	Down-regulated	
		ME/CFS	↑(PBMCs) [74]		
	**miR-143**	FM	↓(CSF*) [67]<br>↓(PBMCs) [69]	Down-regulated	
		ME/CFS	↑(Plasma) [73]		
	**miR-126**	FM	↑(WBC**) [71]	Down-regulated	
		ME/CFS	↓(Plasma) [73]<br>↑(PBMCs) [74]		
	miR-27b	FM	↓(CSF*) [67]	Down-regulated	Bhat-Nakshatri P *et al*., 2009[128]; Tilghman SL *et al*., 2012[129]
		ME/CFS	↑(PBMCs) [74]		
	**let-7g**	FM	↓(CSF*) [67]	Up-regulated	Bhat-Nakshatri P *et al*., 2009[128]
		ME/CFS	↓(Plasma) [73]	Down-regulated	Tilghman SL *et al*., 2012[129]
	**miR-21**	FM	↓(CSF*) [67]<br>↓(PBMCs) [69]	Up-regulated	Bhat-Nakshatri P *et al*., 2009[128]
		ME/CFS	↓(NK cells/ CD8+) [72]	Down-regulated	Wickramasinghe NS et al., 2009[130]; Tilghman SL *et al*., 2012[129]
**Dihydrotestosterone (DHT)**	miR-29a	FM	↓(CSF*) [67]	Up-regulated	Waltering KK *et al*., 2011[131]
	**miR-21**	FM	↓(CSF*) [67]<br>↓(PBMCs) [69]	Up-regulated	
		ME/CFS	↓(NK cells/ CD8+) [72]		
	miR-22	FM	↑(WBC**) [71]	Up-regulated	
		ME/CFS	↑(PBMCs) [74]		
	miR-150*	FM	↓(CSF*) [67]	Down-regulated	
	miR-221	FM	↑(WBC**) [71]	Down-regulated	

**Estrogen**	**miR-143**	FM	↓(CSF*) [67] / ↓(PMBCs) [69]	Down-regulated	Yu X *et al*., 2012[132]
		ME/CFS	↑(Plasma) [73]		
	miR-16	FM	↓(CSF*) [67]	Down-regulated	
		ME/CFS	↓(Plasma) [73]		
**Glucose**	miR-23b	FM	↓(CSF*) [67]	Up-regulated	Feng B *et al*., 2014[133]
	miR-133a	FM	↓(Serum) [70]	Up-regulated	
	miR-150	FM	↓(CSF*) [67]	Up-regulated	
	miR-381	ME/CFS	↑(Plasma) [73]	Up-regulated	
	miR-450b-5p	ME/CFS	↑(Plasma) [73]	Up-regulated	
	**miR-1**	FM	↓(Serum) [70] ↑(WBC**) [71]	Down-regulated	
	miR-34c-5p	FM	↓(CSF*) [67]	Down-regulated	
	miR-182	FM	↓(CSF*) [67]	Down-regulated	
	miR-346	FM	↓(Serum) [70]	Down-regulated	
	miR-526b	FM	↑(CSF*) [67]	Down-regulated	
	miR-616	FM	↑(CSF*) [67]	Down-regulated	
	miR-708	FM	↓(CSF*) [67]	Down-regulated	
	miR-199a-5p	FM	↑(WBC**) [71]	Down-regulated	
		ME/CFS	↑(PBMCs) [74]		
	miR-29a	FM	↓(CSF*) [67]	Down-regulated	Du B *et al*., 2010[134]
	miR-29c	FM	↓(CSF*) [67]	Down-regulated	
	miR-195	FM	↓(CSF*) [67]	Down-regulated	
	miR-374b	FM	↓(Serum) [68]	Down-regulated	
	miR-16	FM	↓(CSF*) [67]	Down-regulated	
		ME/CFS	↓(Plasma) [73]		
**Oleic acid**	miR-181a-5p	ME/CFS	↑(PBMCs) [74]	Up-regulated	Gil-Zamorano J *et al*., 2014[108]
**Palmitic acid**	**miR-1**	FM	↓(Serum) [70] ↑(WBC**) [71]	Up-regulated	Gil-Zamorano J *et al*., 2014[108]
	miR-106b	ME/CFS	↓(NK cells) [72]	Up-regulated	
**Progesterone**	**miR-21**	FM	↓(CSF*) [67] ↓(PBMCs) [69]	Up-regulated	Bae J *et al*., 2012[135]
		ME/CFS	↓(NK cells/ CD8+) [72]		
	miR-27b*	FM	↓(CSF*) [67]	Up-regulated	Cochrane DR *et al*., 2012[136]
		ME/CFS	↑(PBMCs) [74]		
	miR-30c-1*	ME/CFS	↑(PBMCs) [74]	Up-regulated	
	miR-20a	FM	↓(CSF*) [67] ↓(Serum) [70]	Down-regulated	
	**miR-30b**	FM	↓(CSF*) [67] ↓(Serum) [68]	Down-regulated	
	miR-101	FM	↓(CSF*) [67]	Down-regulated	
	miR-130a	FM	↑(WBC**) [71]	Down-regulated	
	miR-301a	FM	↑(WBC**) [71]	Down-regulated	
	miR-29c	FM	↓(CSF*) [67]	Down-regulated	
	miR-19b	FM	↓(CSF*) [67]	Down-regulated	
		ME/CFS	↑(PBMCs) [74]		
	miR-324-5p	ME/CFS	↑(PBMCs) [74]	Down-regulated	

	miR-370	ME/CFS	↑**(Plasma)** [73]	**Down-regulated**	Bae J et al., 2012[135]; Cochrane DR *et al*., 2012[136]
**Testosterone**	miR-29a	FM	↓**(CSF*)** [67]	**Up-regulated**	Wang WL *et al*., 2011[137]
**Vitamin D3**	let-7a	FM	↓**(Serum)** [68]	**Up-regulated**	Jorde R *et al*., 2012[138]
	let-7d	FM	↑**(WBC**)** [71]	**Up-regulated**	
	miR-221	FM	↑**(WBC**)** [71]	**Up-regulated**	
	miR-374b	FM	↓**(Serum)** [68]	**Up-regulated**	
	miR-338-3p	FM	↓**(PBMCs)** [69]	**Up-regulated**	
	miR-99b	FM	↓**(CSF*)** [67]	**Up-regulated**	
		ME/CFS	↑**(PBMCs)** [74]		
	miR-26a	FM	↓**(CSF*)** [67]	**Up-regulated**	
		ME/CFS	↓**(Plasma)** [73]		
	miR-146a	FM	↑**(WBC**)** [71]	**Up-regulated**	
		ME/CFS	↓**(NK cells)** [72]		
	**miR-191**	ME/CFS	↓**(NK cells)** [72]<br>↑**(PBMCs)** [74]	**Up-regulated**	
	miR-331-3p	FM	↑**(WBC**)** [71]	**Up-regulated**	
		ME/CFS	↑**(Plasma)** [73]		
	miR-339-5p	FM	↑**(WBC**)** [71]	**Up-regulated**	
		ME/CFS	↑**(WBC**)** [71]		
	miR-151-3p	ME/CFS	↑**(PBMCs)** [74]	**Up-regulated**	
	**miR-424**	FM	↓**(CSF*)** [67]<br>↑**(Serum)** [70]	**Down-regulated**	
	miR-22	FM	↑**(WBC**)** [71]	**Down-regulated**	
		ME/CFS	↑**(PBMCs)** [74]		
	miR-324-5p	ME/CFS	↑**(PBMCs)** [74]	**Down-regulated**	
	miR-106b	ME/CFS	↓**(NK cells)** [72]	**Down-regulated**	

Bolded miRs correspond to miRs DE according to more than one FM or ME/CFS study. Underlined miRs correspond to miRs DE in both, FM and ME/CFS studies.
